# Study on small ruminant brucellosis and owners awareness in two selected districts of southern region, Ethiopia

**DOI:** 10.1002/vms3.992

**Published:** 2022-11-11

**Authors:** Desalegn Dosa, Nejib Mohammed, Mesfin Mathewos

**Affiliations:** ^1^ Sodo Regional Veterinary Laboratory Wolaita Sodo Ethiopia; ^2^ College of Agriculture Arbaminch University Arbaminch Ethiopia; ^3^ School of Veterinary Medicine Wolaita Sodo University Wolaita Sodo Ethiopia

**Keywords:** brucellosis, complement fixation test, Ethiopia, Rose Bengal plate test, seroprevalence, small ruminant

## Abstract

**Introduction:**

Brucellosis is one of the infectious diseases that has the greatest impact on the productivity of sheep and goats. A cross‐sectional study followed by a simple random sampling technique was used to investigate the seroprevalence of brucellosis (Rose Bengal plate test; RBPT and complement fixation test; CFT) in small ruminants and its related risk variables from November 2019 to June 2020 in Kolme and Abala Abaya districts. A questionnaire was also given to owners to assess their existing knowledge of the disease.

**Result:**

Using the RBPT and CFT, 28 (4.1%) and 23 (3.33%) of the 690 animals were found to be seropositive for brucellosis, respectively. In this study, the seroprevalence of brucellosis detected in the Kolme district (5.3%) was greater when compared to Abala Abaya (1.0%). The odds of *Brucella* infection were greater for goats (odds ratio [OR] 6.0, 95% confidence interval [CI] 16 0.8–44.9) than for sheep. The odds of adult animals (OR 0.05, 95% CI 0.03–0.07) being positive for brucellosis was higher than young animals. A statistically significant difference in the seropositivity of brucellosis was detected in univariate logistic regression among districts, different age groups, herd size, parity number, and reproductive health problems except for species and sex, but in multivariate logistic regression, only reproductive health problems were revealed a statistically significant difference. Out of 138 families, 100% of respondents were unaware of brucellosis, 94.5% drink raw milk, and 74% handle animals with retained fetal membranes with their bare hands.

**Conclusion:**

This study showed that brucellosis was a widely spread disease in the study areas and poses a substantial public health danger. To reduce the spread of the disease in small ruminants, public health risks, and economic losses, stringent vaccination application and awareness of personal hygiene are critical.

## Graphical abstract

Brucellosis is one of the infectious illnesses that have the greatest impact on the productivity of sheep and goats.

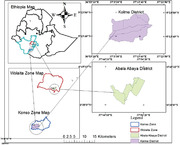

## 1 INTRODUCTION

Goats and sheep are major domestic animals in African tropical livestock production systems, accounting for 21% of the global small ruminant population (Ashenafi et al., 2007). Ethiopia is one of East Africa's developing countries, with 31.30 million sheep and 32.74 million goats primarily raised in the country's lowland and pastoral regions (Addis & Desalegn, 2018). Because of their high proliferation rate and great ability to adapt to various environmental conditions, these small ruminants represent an important export commodity that significantly contributes to the livelihood and national economy of rural farmers as a source of food (milk and meat), wool, skins, source of income, and monetary asset, especially in pastoral and lowland areas of Ethiopia (Adams et al., 2021). Small ruminants are a vast resource; however, production from this valuable asset is not fully realized due to several technical and non‐technical factors, including nutritional deficiency, husbandry difficulties, water scarcity, inadequate marketing, and losses associated with infectious diseases, primarily brucellosis (Ashenafi et al., 2007; Geletu et al., 2021; Tewodros & Dawit, 2015).

Brucellosis is a zoonotic disease (Franc et al., 2018) that is caused by microscopic coccobacilli bacteria of the genus *Brucella*. This Gram‐negative bacterium is slow growing and capable of surviving and replicating within epithelial cells, placental trophoblasts, dendritic cells, and macrophages (Tekle et al., 2019).

In goats and sheep, *Brucella melitensis* produces the disease; however, in cattle, *Brucella abortus* can cause clinical brucellosis, and in rams, *Brucella ovis* causes epididymitis (Kusiluka & Kambarage, 1996). The disease affects the animal's reproductive system, causing significant production and productivity losses such as decreased milk production, abortion, stillbirth, acute orchitis, weak offspring, weight loss, culling, and condemnation of infected animals due to infertility, lameness, and trade and export restrictions (Makita et al., 2011).

In endemic areas, brucellosis is primarily transmitted between animals through direct contact with infected animals or contact with an environment that has been contaminated with infected birthing tissues or fluids, including the placenta, aborted fetuses, or uterine discharge. Humans most frequently acquire brucellosis by consuming infected raw animal products (raw, unpasteurized milk, and soft cheeses) but can also develop the disease following contact with infected animal tissues or secretions (especially unpasteurized milk and soft cheeses) (Gupte & Kaur, 2015; Radostits et al., 2006).

Different species of *Brucella* and their biovars has distinct distributions. The bacterium *B. abortus* can be found all over the world. *Brucella melitensis* and *Brucella suis* have a sporadic distribution (Acha & Szyfres, 2003). The disease's occurrence in animals is related to a variety of factors. Animals' age, sex, and reproductive status are all host variables that can influence their susceptibility to infection (Nicoletti, 2010). Farmers, veterinarians, and others in the livestock industry are at high risk of contracting the disease (Sintayehu et al., 2015; Tewodros & Dawit, 2015).

The World Health Organization has designated brucellosis as one of the world's main ‘neglected zoonotic diseases’, owing to the disease's disproportionate impact on low‐income countries (Franc et al., 2018). In Ethiopia, the first case of brucellosis was reported almost 50 years ago (Domenech & Lefevre, 1974). Many studies detailing the prevalence and associated risk factors of small ruminant brucellosis throughout different parts of Ethiopia have been reported (Ashagrie et al., 2011; Asmare et al., 2013; Ferede et al., 2011; Melese, 2016; Sintayehu et al., 2015; Teshale et al., 2006; Tewodros & Dawit, 2015; Yesufa et al., 2011). Ethiopia is a large country with a huge livestock population, and previously published work has only described seroprevalence for specific areas and not the whole country (Tamrat et al., 2020). Furthermore, no published research work on small ruminant brucellosis was found in the current study region. Therefore, this study was initiated to investigate the seroprevalence and the associated risk factors of small ruminant brucellosis in the Kolme and Abala Abaya districts.

## 2 MATERIALS AND METHODS

### 2.1 Study area

**FIGURE 1:**
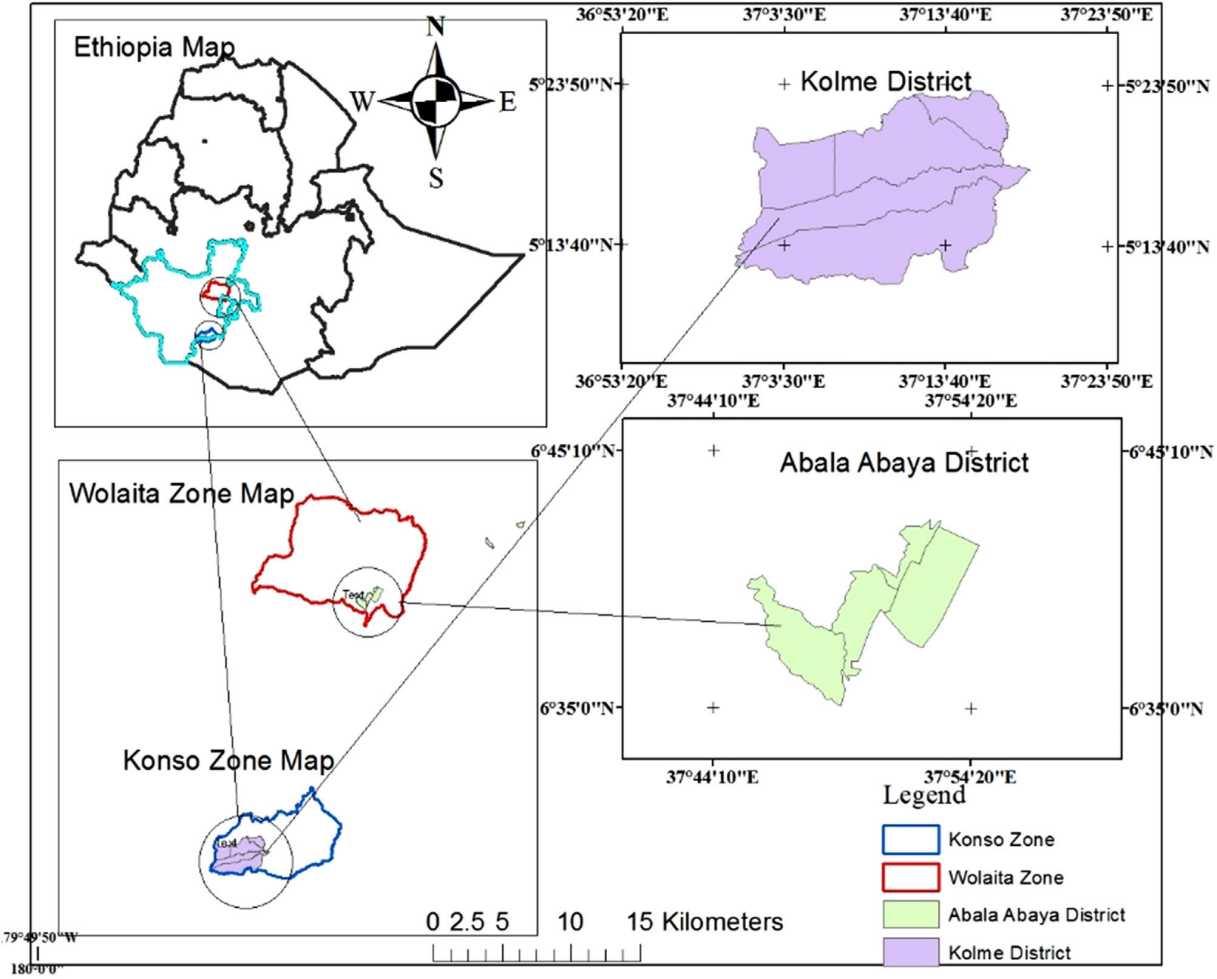
Map of the Kolme and Abala Abaya districts

The study was undertaken in the Southern Nation Nationalities Peoples (SNNP) Region in Kolme and Abala Abaya districts from December 2019 to May 2020. The districts of Kolme and Abala Abaya are about 600 and 345 km south of Addis Ababa, respectively (Figure 1). Geographically, the Abala Abaya district lies between 37° 44' 10" and 37° 54' 20" E longitudes and 6° 35' 0" to 6° 45' 10" N latitudes, whereas the Kolme district lies between 36o 53' 20" and 37o 23' 50" E longitudes and 5o 13' 40" to 5o 23' 50" N latitudes. In the Kolme area, the altitude ranges from 500 to 2500 m above sea level, whereas in the Abala Abaya district, the altitude ranges from 700 to 2275 meters. The annual average rainfall in the area was reported as 570 mm at Kolme and 854 mm in Abala Abaya, according to the local meteorological station, while the average annual temperature in the Abala Abaya district was 31.59°C (21–27.5°C in sub‐zone ranges), and the temperature typically varies between 17.22 and 35.55°C throughout the year in Kolme, with temperatures rarely falling below 15.55°C or rising above 37.77°C. Agro‐pastoralism in Kolme and the lowland mixed agricultural system in Abala Abaya are the main economic activities in the research area. Cattle are the most common animal species in terms of population number, followed by small ruminants. According to each woreda Livestock and Fishier Office report, Kolme district had 140,834 goats and 75,804 sheep, whereas Abala Abaya district had 25,000 sheep and 53,706 goats (Ashenafi et al., 2007).

### 2.2 Study populations

This study focused on indigenous sheep and goats in the Kolme and Abala Abaya districts, which were kept in the extensive production system. The study comprised sheep and goats aged 6 months and above, as well as both sexes in the selected flock that had never been vaccinated against brucellosis. Information regarding putative risk factors including origins of the animal, species, sex category, parity, and reproductive health concerns (abortion, stillbirth, retained fetal membrane) was documented as previously described (Abebe & Yami, 2008; Shively, 1985). The age of sheep and goats was determined based on their dental eruption patterns. Animals were classified as young (1–3 years old) if they had four or fewer permanent teeth and adult (4–5 years old) if they had more than four permanent teeth (Steel, 1996; Vatta et al., 2006).

From December 2019 to May 2020, a cross‐sectional study was carried out in the Kolme and Abala Abaya districts to investigate the seroprevalence and the associated risk factors of small ruminant brucellosis.

### 2.3 Sample size determination

The sample size was calculated according to the approach suggested by Thrusfield (2018). The seroprevalence of small ruminant brucellosis was estimated at 5.9% using previously reported results in districts near those studied herein (Melese, 2016; Wakene et al. 2017). Six hundred and ninety goats and sheep were sampled in this study, which is twice as large as the sample size calculated to achieve a level of significance of 0.05% and a 95% level of confidence. Thus, 345 goats and sheep were chosen as the overall sample size. However, the sample size was enlarged two times to maximize precision, resulting in 690 goats and sheep being sampled for the seroprevalence investigation (Thrusfield, 2018).

### 2.4 Sampling techniques and sample collection

A multistage sampling technique was carried out to select the zone from the southern region. Among different zones of the region, the zones (Jinka and Wolaita) and districts (Kolme and Abala Abaya districts) were selected purposively based on the population density of sheep and goats. Peasant association: three from Kolme (Masoya, Abaya, and Gete) and three from Abala Abaya (Abala Maraka, Abala Sipa, and Abala Faracho), households, and the herd were selected randomly from both districts. The households that have an average animal number of 10 in Abala Abaya districts and 30 in Kolme districts were included to collect the sample. Accordingly, a total of 690 small ruminants (*n* = 546 goats and *n* = 144 sheep) were sampled.

After handling the animal carefully, about 10 ml of blood was obtained aseptically from the jugular vein of each animal using plain vacutainer tubes. Using a permanent marker, all samples were serially identified and correctly labelled. The blood sample was spun at 3000 rpm for 4 min before being gently split into 2 ml cryovials tubes and delivered to the Sodo Regional Veterinary Laboratory using an icebox. The Rose Bengal plate test (RBPT) was used to screen sera samples. After using the RBPT test, all positive samples were sent to the National Veterinary Institute for confirmation using the complement fixation test (CFT) (OIE, 2018).

#### 2.4.1 Rose Bengal plate test

The whole serum sample was first examined using the RBPT, which was done by adding 25 μl of antigen and 75 μl of serum onto a plate. The antigen and test serum were then completely mixed with the plastic applicator, shaken for 4 min, and the degree of agglutination was visually inspected and recorded as positive or negative for the presence or absence of agglutination (OIE, 2009). The degree of agglutination was then recorded as 0, +, ++, +++, with 0 indicating no agglutination, + indicating barely visible agglutination, ++ indicating fine agglutination, and +++ indicating coarse clumping. The samples with no agglutination (0) were considered negative, whereas those with +, ++, and +++ were considered positive and recorded (Ashenafi et al., 2007; Rose & Roepke, 1957).

#### 2.4.2 Complement fixation test

The CFT was used to confirm the results of those sera that tested positive by the RBPT. Anti‐*Brucella* antibodies were detected in the serum using standard *B. abortus* antigen for CFT (from the Veterinary Laboratories Agency, Addlestone, United Kingdom). The control sera and complement were obtained from the German Federal Institute for Consumer and Veterinary Medicine in Berlin. The minimal positive threshold was set at a 25% haemolytic response at a dilution of 1:5. Sera with a strong reaction of approximately 100% fixation of the complement (4+) at a dilution of 1:5, sera with about 75% fixation of the complement (3+) at a dilution of 1:10, sera with about 50% fixation of the complement (2+) at a dilution of 1:20, and sera with about 25% fixation at a dilution of 1:40 (+) will be classified as positive (Ashenafi et al., 2007).

#### 2.4.3 Questionnaire survey

A pre‐tested semi‐structured questionnaire was given to animal owners to know their knowledge about brucellosis, consuming raw animal products (milk, blood, or meat), handling aborted fetuses and retained placentas by bare hand, reproductive health problems, isolation of diseased animals from the herd, herd size, and history of contact with another herd. As a result, 138 households (90 from Kolme and 48 from Abala Abaya) took part in the research. The overall sample size for a household interview was calculated according to Cochran (Cochran, 1977).

### 2.5 Data analysis

A Microsoft Excel spreadsheet was used to store the information gathered from the questionnaire and laboratory testing. Following coding and editing, STATA/IC 13 was used to do the appropriate statistical analysis (2013, College Station). The frequency and proportion of both dependent and independent variables were determined using descriptive statistics. The *χ* ^2^ test was also employed to see if there was a significant difference between the independent variables (origin of animals, species, herd size, sex, age, parity, and reproductive health problems) (abortion, stillbirth, and RFM) with expected outcomes (brucellosis). Using univariate logistic regression analysis, the different possible risk factors were evaluated for their association with the seroprevalence of brucellosis in small ruminants. To develop the likely model (*p* < 0.05), non‐collinear variables with *p*‐value ≤0.25 were all subjected to multivariable logistic regression analysis (Dohoo et al., 2003).

## 3 RESULT

### 3.1 Overall seroprevalence of small ruminant brucellosis

The seroprevalence of brucellosis as detected using the RBPT and CFT for each district and kebeles was presented in Table 1. The seroprevalence of *Brucella* infection in Kolme (5.3%) was greater than in Abala Abaya districts (1.0%) and has shown a statistically significant difference (*p* < 0.05).

**TABLE 1:** The overall seroprevalence of small ruminant brucellosis in districts and each kebeles

District	Kebele	Number of sera	RBPT positive (%)	CFT positive (%)	*χ* ^2^	*p*‐Value
Kolme	Masoya	180	23 (12.7)	20 (11.1)	47.7	0.0001
	Abaya	100	0 (0)	0(0)		
	Gete	100	0 (0)	0 (0)		
		380	23 (6.1)	20 (5.3)		
Abala Abaya	Abala Maraka	110	5 (4.5)	3 (2.7)		
	Abala Sipa	77	0 (0)	0 (0)		
	Abala Faracho	123	0 (0)	0 (0)		
	Three	310	5 (1.6)	3 (1.0)		
Total	Six	690	28 (4.1)	23 (3.33)		

Abbreviations: CFT, complement fixation test; RBPT, Rose Bengal plate test.

### 3.2 Association of risk factors with seroprevalence of small ruminant brucellosis

When using the CFT, goats had a greater seroprevalence of brucellosis (4.0%) than sheep (0.7%). Except for sex, which showed an insignificant association (*p* > 0.05) with the seroprevalence brucellosis, a statistically significant difference (*p* < 0.05) was identified with the occurrence of brucellosis among all other risk variables. Females had a greater seroprevalence of brucellosis (4.0%) than males (1.2%). Only 4.4% of small ruminants in the adult age group were found to be seropositive. Furthermore, seroprevalence was found to be greater in herd sizes >30 (4.0%) and parity >4 (7.3%) when compared to their respective categories (Table 2).

**TABLE 2:** Association of risk factors with brucellosis reactivity in small ruminants

Variable	Category	No. of sera examined	No. of RBPT positive sera (%)	No. of CFT positive sera (%)	*χ* ^2^	*p*‐Value
Districts	Kolme	380	23 (6.1)	20 (5.3)	9.77	0.002
	Abala Abaya	310	5 (1.6)	3 (1.0)		
Species	Caprine	546	25 (4.6)	22 (4.0)	3.93	0.047
	Ovine	144	3 (2.1)	1 (0.7)		
Herd size	1–10 (small)	44	0 (0)	0 (0)	13.3	0.001
	11–30 (medium)	272	4 (1.5)	2 (0.74)		
	>30 (large)	374	24 (6.4)	21 (5.6)		
Sex	Female	525	25 (4.8)	21 (4.0)	3.03	0.082
	Male	165	3 (1.8)	2 (1.2)		
Age	Adult	528	28 (5.3)	23 (4.4)	7.3	0.007
	Young	162	0 (0)	0 (0)		
Parity	0	146	4 (2.7)	2 (1.4)	13.2	0.001
	1–3	255	15 (5.88)	13 (5.1)		
	>4	124	9 (7.3)	9 (7.3)		
Abortion	Yes	62	16 (25.8)	15 (23.8)	91.9	0.001
	No	317	12 (3.7)	8 (2.5)		
Stillbirth	Yes	21	3 (14.3)	3 (14.3)	8.1	0.005
	No	358	25 (6.9)	20 (5.5)		
RFM	Yes	16	10 (62.5)	9 (56.25)	152.8	0.001
	No	363	18 (4.9)	14 (3.8)		

Abbreviations: CFT, complement fixation test; RBPT, Rose Bengal plate test; RFM, retained fetal membrane.

### 3.3 Univariable logistic regression analysis of potential risk factors

On univariable logistic regression analysis, associated risk factors such as district, herd size, parity, age, and history of reproductive health problems (abortion, stillbirth, and retained fetal membrane) had a statistically significant effect on seropositivity (*p* < 0.05), whereas small ruminant species and sex had no statistically significant effect on seropositivity (*p* > 0.05) (Table 3).

**TABLE 3:** Univariable logistic regression analysis of potential risk factors on complement fixation test result

Risk factor	Category	No. of animals tested	No. of positive	Prevalence (%)	*p*‐Value	OR (95% CI)
District	Kolme	380	20	5.3	0.005	5.68 (1.67–19.31)
	Abala Abaya	310	3	1.0		
Species	Goats	546	22	4.03	0.081	6.0 (0.8–44.9)
	Sheep	144	1	0.7	‐	
Herd size	Small	44	‐	‐	‐	‐
	Medium	272	2	0.7	0.004	7.96 (0.04–0.85)
	Large	374	21	5.6	‐	
Sex	Female	525	21	4.0	0.101	3.39 (0.79–14.64)
	Male	165	2	1.2	‐	
Age	Young	162	‐	‐	‐	‐
	Adult	528	23	4.4	0.001	0.05 (0.03–0.07)
Parity	Null	146	2	1.4	‐	‐
	1–3	255	13	5.1	0.006	8.3 (1.85–37.13)
	≥4	124	8	6.5	0.003	10.6 (2.23–50.91)
Abortion	Yes	62	15	23.8	0.01	24.3 (9–61.32)
	No	317	8	2.5		
Stillbirth	Yes	21	3	14.3	0.02	4.5 (1.22–16.67)
	No	358	20	5.5		
RFM	Yes	16	9	56.25	0.01	62.2 (19.1–202.8)
	No	363	14	3.8		

Abbreviations: CI, confidence interval; OR, odds ratio; RFM, retained fetal membrane.

### 3.4 Multivariable logistic regression analysis of potential risk factors

Variables with a *p*‐value ≤0.25 were all subjected to multivariable logistic regression analysis to construct the likely model (*p* < 0.05). As a result, multivariable logistic regression analysis employing this model demonstrated a statistically significant association between the prevalence of small ruminant brucellosis with history of abortion, stillbirth, and retained fetal membrane (Table 4).

**TABLE 4:** Multivariable logistic regression analysis of potential risk factors

Risk factor	Category	No. of animals tested	No. of positive	Prevalence (%)	*p*‐Value	OR (95% CI)
Abortion	Yes	62	15	23.8	0.001	11.4 (3.5–37.6)
	No	317	8	2.5		
Stillbirth	Yes	21	3	14.3	0.005	9 (2–41)
	No	358	20	5.5		
RFM	Yes	16	9	56.25	0.001	15 (3.8–60.5)
	No	363	14	3.8		

Abbreviations: CI, confidence interval; OR, odds ratio; RFM, retained fetal membrane.

### 3.5 Assessment of Awareness among Small Ruminants Owners’

**FIGURE 2:**
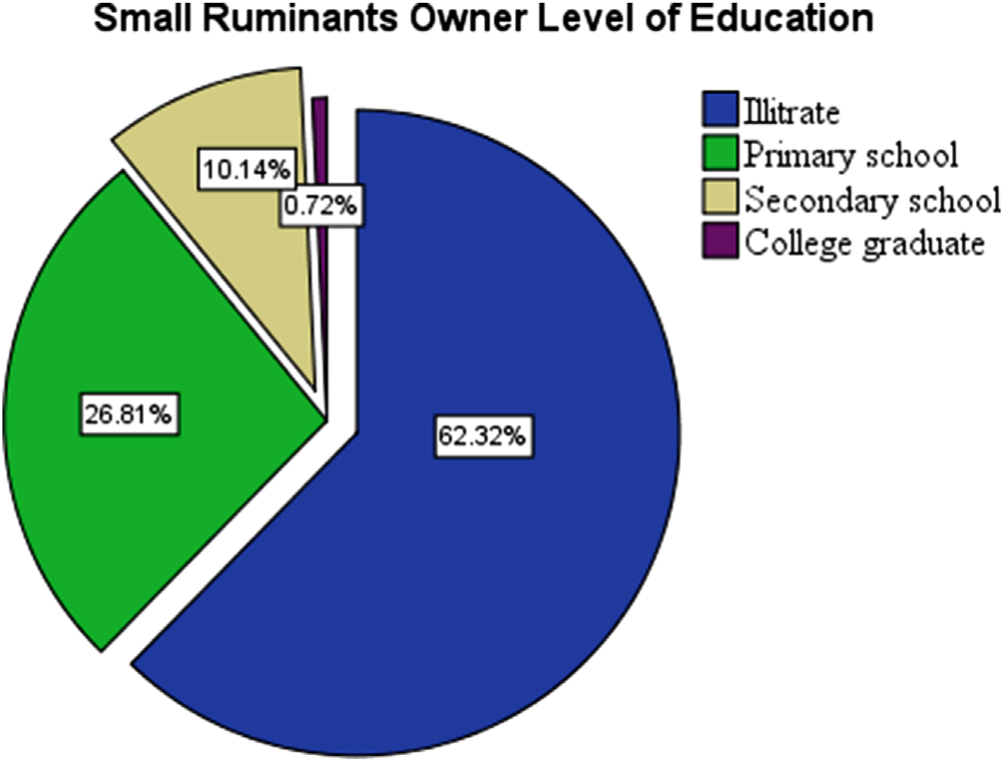
Level of education of questionnaire respondents

The research areas’ household educational level revealed that the majority of small ruminant owners interviewed (62.32%) were illiterate (Figure 2).

All of the respondents (100%) were unaware of the *Brucella* infection. Around 79.7% of owners used their bare hands to help their animals during parturition which exposed them to the disease. Because of a lack of information about disease transmission, 67.4% of animal owners did not separate sick animals from healthy ones. Approximately 57.2% of respondents’ herds had experienced reproductive health problems such as abortion, stillbirth, or retained fetal membrane, and 75.4% of respondents’ herds have had previous contact with other herds during feeding and watering time. Note that 93.5% of those polled said that they ate raw animal products regularly. The majority of households in this study were unable to implement any control measures in response to the widespread abortion in small ruminants. Furthermore, they were uninformed about the illnesses’ zoonotic potential (Table 5).

**TABLE 5:** Results of a questionnaire survey on the awareness, knowledge, and disease management practices of rural farmers on small ruminant brucellosis

Risk factors	No. of respondents (%)		Risk factors	No. of respondents (%)	
Know about brucellosis	Yes	0 (0%)	Contact with other herds	Yes	104 (75.4%)
	No	138 (100%)		No	34 (24.6%)
Use protective gloves while supporting animals during parturition	Yes	28 (20.3%)			
	No	110 (79.7%)	Flock size	1–10 (small)	38 (27.5%)
Consume raw animal products (milk, blood, or meat)	Yes	129 (93.5%)		11–30 (medium)	43 (31.2%)
	No	9 (6.5)		>30 (large)	57 (41.3%)
Handle aborted fetus and retained placenta by bare hand	Yes	102 (74%)			
	No	36 (26%)	Cause of RHP	Mechanical	10 (7.2%)
Reproductive health problem	Yes	79 (57.3%)		Infection	65 (47.2%)
	No	59 (42.7%)		Poison	0 (0%)
Isolation of diseased animals from the herd	Yes	45 (32.6%)		Unknown	63 (45.6%)
	No	93 (67.4%)			

Abbreviation: RHP, reproductive health problem.

## 4 DISCUSSION

Out of 690 (546 goats and 144 sheep) sera samples tested for small ruminant brucellosis in the Kolme and Abala Abaya districts, 28 (4.1%) tested positive on the RBPT, and 23 (3.33%) tested positive for CFT for brucellosis. The high seroprevalence of *Brucella* infection when using the RBPT was in line with the previous reports of Ashagrie et al. (2011) who indicated a seroprevalence of 4.2%. Contrary to our findings, a lower seroprevalence of *Brucella* infection in Ethiopia ranging from 0.7% to 3.7% has been previously reported (Ashenafi et al., 2007; Asmare et al., 2013; Ferede et al., 2011; Habtamu et al., 2015; Sintayehu et al., 2015; Teshale et al., 2006; Tewodros & Dawit, 2015). Furthermore, the prevalence ranges from 1.6% to 14.6% and 1.7% to 16.45% in sheep and goats, respectively, in Afar and Somali regions (Ashenafi et al., 2007; Teshale et al., 2006).

The overall seroprevalence of brucellosis in CFT (3.3%) was found to agree with the previous report (Melese, 2016; Sintayehu et al., 2015; Teklue et al., 2013). In contrast, a higher overall seroprevalence was previously described by Wakene et al. (2017) in northern parts of Ethiopia. The CFT test is commonly used as a confirmatory test because of its high specificity (Padilla Poester et al., 2010). When using this test, the number of positive animals declined from 28 to 23 with a false‐positive result of 5/28 (17.8%). This is likely due to the CFT reducing the occurrence of false positives due to cross‐reacting bacteria that can occur with RBPT. Indeed, the RBPT is susceptible to cross‐reaction with other Gram‐negative bacteria such as *Yersinia enterocolitica*, O:9, *Escherichia coli*, O:157, and some *Salmonella* spp (Garin‐Bastuji et al., 2006; OIE, 2009).

The seroprevalence of *Brucella* infection in Kolme and Abala Abaya districts showed a statistically significant difference (*p* < 0.05) with the occurrence of brucellosis. Similar findings have been previously reported from different parts of the country (Ashenafi et al., 2007; Deddefo et al., 2015; Mengistu, 2007; Tigist et al., 2011; Yibeltal, 2005). The difference in the seroprevalence of *Brucella* infection could be associated with unrestricted movement of animals for searching pasture and water nearby watering points, especially during the dry season, environmental factors, breed differences, sample size, and the tests used.

A statistically significant difference in the seropositivity of *Brucella* infection was observed between species. The seroprevalence of *Brucella* infection in goats (4.03%) was higher than in sheep (0.7%). Similar to this finding, a higher seroprevalence of *Brucella* infection ranging from 15.4% to 1.9% has been previously reported (Adugna et al., 2013; Ashenafi et al., 2007; Bekele et al., 2011; Mengistu, 2007; Teshome et al., 2018; Wedajo et al., 2015) in goats and sheep. On contrary, Deddefo et al. (2015) in Arsi and East Shoa recorded a higher seroprevalence of brucellosis in goats (4.9%) and sheep (4.4%) with no statistically significant difference. This difference might be due to the differences in flock sizes and the number of goats and sheep kept in the herd. Most breeds of goats are fully susceptible, but the susceptibility of sheep breeds differs widely (Radostitis et al., 2007). The breed‐specific difference in the case of goats may be due to persistent infection of mammary glands and supramammary lymph nodes because of constant or intermittent shedding of organisms within the milk during succeeding lactations. However, in the case of sheep, the differences may due to the self‐limiting nature of the disease which is seldom accompanied by prolonged excretion of the bacteria (Radostitis et al., 2007).

The seropositivity of *Brucella* infection between sex was not revealed as a statistically significant difference as previously described by Ashenafi et al. (2007). The lack of statistically significant difference between sexes could be due to the smaller sample size of males, and the habit of keeping males in the herd for a shorter period may decrease their exposure to the disease. Yet, Adugna et al. (2013) have also reported that males are less susceptible to *Brucella* infection due to the absence of erythritol.

A statistically significant difference that was observed among age groups with the occurrence of brucellosis was found to be in line with Radostits et al. (2006) and Quinn et al. (2004) who have reported that brucellosis is essentially a disease of sexually mature animals. However, young animals may acquire latent infections even though they are more resistant to *Brucella* infection as they frequently clear an established infection (Walker, 1999). This is due to sex hormones and erythritol, which stimulate the growth and multiplication of *Brucella* organisms, which tend to increase in concentration with age and sexual maturity (Radostits et al., 2000).

In this study, a statistically significant difference (*p* < 0.05) was observed between herd‐level prevalence and individual animal‐level prevalence of brucellosis in the Kolme and Abala Abaya districts. This result was in agreement with the report of Wakene et al. (2017) and Adugna et al. (2013) who appreciated a high seroprevalence of brucellosis at the herd level but contrary to Mohammed et‐al. (2017) and Ferede et al. (2011). Although a statistically significant association was observed as parity increased with the seropositivity of brucellosis. This was compatible with Melese (2016), Ashagrie et‐al. (2011), and Deddefo et al. (2015). This difference could be due to the effect of reproductive physiology and hormone on the genetic performance of individual animals and differences in the animal population in their respective niche. Furthermore, ewes having a history of abortion, stillbirth, and retained fetal membranes have revealed a statistically significant difference with the seroprevalence of brucellosis. This finding was in agreement with the report of Wakene et al. (Wakene et al., 2017) but contrary to Deddefo et al. (2015) and Adugna et al. (2013). The difference might be due to the effect of the reproductive and sexual hormones on the pathogenesis of the disease in small ruminants (Radostits et al., 2000).

Among small ruminant owners, 62.32% were illiterate. However, 100% of owners were unaware of the disease, and 79.7% of them used their bare hands during parturition. This was in agreement with the report of Wakene et al. (2017). This study also revealed that 67.4% of owners are not aware of the transmission of brucellosis through contact with other herds during feeding and watering times, and consumption of raw animal products, so they have not practiced the isolation of healthy animals from diseased ones. Approximately 57.2% of respondents’ herds had experienced reproductive health problems such as abortion, stillbirth, or retained fetal membrane due to a lack of using any control measure to prevent abortion in small ruminants. Hence, this is the most common means of exposure of the rural community to the disease in endemic areas as reported by Olsen and Palmer (2014).

Brucellosis is a severe health danger for the entire community due to close contact between humans and their animals, which sometimes share the same housing enclosures. In Ethiopia, reproductive issues such as abortion, stillbirth, and retained fetal membranes are the most common causes of reproductive wastage in small ruminants, and humans may become infected while handling infected material. It has yet to be determined if brucellosis or malaria is the source of human illness with symptoms such as fever, aches and pains, weariness, and debility. Consequently, there is a high risk of human brucellosis in the study area. This outcome is in agreement with the study conducted by Ashenafi et al. (2007) and Habtamu et al. (2015).

## 5 CONCLUSION AND RECOMMENDATIONS

This study revealed a high seroprevalence of brucellosis in goats as compared to sheep in Kolme and Abaya Abala districts. All potential risk factors, including district, herd size, parity, age, and a history of reproductive health problems (abortion, stillbirth, and retained fetal membrane), have shown a statistically significant difference with the occurrence of small ruminant brucellosis. In the current study area, smallholder farmers were found to be at high risk of acquiring *Brucella* infection due to a tradition of ingesting raw milk, traditional animal husbandry practices, handling animals with reproductive difficulties with their bare hands, and lack awareness on the zoonotic nature of the disease. As a result, improved management systems and the implementation of appropriate control measures in the smallholder production system are recommended in the study areas. Further epidemiological research, as well as the identification and isolation of the *Brucella* biotype responsible for the infection, should be carried out.

## AUTHOR CONTRIBUTIONS

All authors made substantial contributions to conception and design, acquisition of data, or analysis and interpretation of data; took part in drafting the article or revising it critically for important intellectual content; agreed to submit to the current journal; gave final approval of the version to be published; and agree to be accountable for all aspects of the work.

## CONFLICTS OF INTEREST

All authors declare conflict of interest.

## FUNDING INFORMATION

This work was not supported by any funding source or institution.

### PEER REVIEW

I would not like my name to appear with my report on Publons https://publons.com/publon/10.1002/vms3.992.

## ETHICS STATEMENT

The Arbaminch University of Research Ethics and Review Committee provided ethical approval for this research. Before collecting blood samples, verbal consent was also sought from owners to take samples from their sheep and goats following strict hygienic measures. The best practice guidelines for animal care were followed as to the purpose of the study, and that the Arbaminch University of Research Ethics and Review Committee approved the verbally informed consent process.

## Data Availability

The datasets used and analyzed during the current study will be available from the corresponding author on reasonable request.
